# Pleiotropy Complicates Human Gene Editing: CCR5Δ32 and Beyond

**DOI:** 10.3389/fgene.2019.00669

**Published:** 2019-07-30

**Authors:** Ting Li, Xia Shen

**Affiliations:** ^1^Biostatistics Group, State Key Laboratory of Biocontrol, School of Life Sciences, Sun Yat-sen Univeristy, Guangzhou, China; ^2^Center for Global Health Research, Usher Institute of Population Health Sciences and Informatics, University of Edinburgh, Edinburgh, United Kingdom; ^3^Department of Medical Epidemiology and Biostatistics, Karolinska Institutet, Stockholm, Sweden

**Keywords:** gene editing, CRISPR, CCR5Δ32, pleiotropy, UK Biobank, autoimmune disease, cardiovascular disease

## Introduction

Half a year ago, Chinese scientist He Jiankui pushed an ethical boundary by claiming to have treated two female infants for potential future HIV infection by altering a small piece of their genome. He was thereafter listed among *Nature*’s 10 people who mattered in 2018. “He was widely criticized for ignoring important ethical considerations and exposing the girls to unknown risks for an uncertain benefit”, as reported by *Nature* (Vol. 564, page 329).

The gene editing target that He Jiankui chose was from a study with participants of European ancestry, wherein in a cohort of HIV-1-infected individuals, none was found to be homozygote for the *CCR5*Δ*32* deletion, despite its relatively high allele frequency (9.2%) in the European population (Samson et al., 1996). Later studies further showed that stem cell transplantation from *CCR5*Δ*32* homozygotes can treat HIV-1-infected individuals (Hütter et al., 2009; Gupta et al., 2019). Thus, introducing the deletion of the *CCR5* gene seems to be protective against HIV-1 infection. However, the potential side effects of the deletion are far from clear.

He Jiankui was criticized for putting the young girls into *unknown* risks. Cyranoski (2018) timely pointed out in *Nature* News that the target variant was reported to have negative effects on a range of human traits. Later, Lander et al. (2019) commented in the same journal to highlight and discuss the medical, scientific, and ethical considerations of gene editing in humans, where they pointed out that the long-term effects on genetically correlated traits need to be understood before performing gene editing on humans.

According to literature, except for documented side effects on, e.g., West Nile virus infection (Glass et al., 2006), celiac disease, and autoimmune thyroid disorders in patients with type 1 diabetes (Słomiński et al., 2017), *CCR5* loss of function was actually reported to be favorable for multiple sclerosis (Barcellos et al., 2000; Kantor et al., 2003), spontaneous hepatitis C viral clearance (Goulding et al., 2005), and chronic and aggressive periodontitis (Cavalla et al., 2018). Although *CCR5* is clearly involved in the human immune system, it is hard to assess its potential side effects.

Very recently, Wei and Nielsen (2019) reported an assessment of CCR5Δ32 homozygote carriers in UK Biobank, who were shown to suffer from 21% increase in their mortality rate. Wei and Nielsen predicted that this Δ32 mutation could be highly pleiotropic and potentially increase the susceptibility to other common diseases.

Here, from a quantitative genetics perspective, we aim to use UK Biobank as a unified source of genomic big data to investigate additional evidence of the substantial pleiotropy of disease-associated DNA variants, starting from the *CCR5* gene that He Jiankui tried to edit using CRISPR.

## Analysis

### *CCR5*Δ*32* Does More Harm Than Good According to UK Biobank

We first focused on the *CCR5*Δ*32* variant that was imputed with quality (variant 3:46414943_TACAGTCAGTATCAATTCTGGAAGAATTTCCAG_T, info score 0.838) in the UK Biobank cohort. This deletion variant was also what was aimed for by He Jiankui in his gene editing surgery, as the variant was documented to prevent the homozygote carriers from HIV infection (Hütter et al., 2009). The association analysis results between this variant and 131 curated disease phenotypes with at least 1000 cases was extracted from the UK Biobank round 2 genome-wide association study (GWAS) results released by Neale’s lab (http://www.nealelab.is/uk-biobank/ukbround2announcement; **Supplementary Table 1**).

The GWAS by Neale’s lab was conducted *via* a simple linear regression of each binary disease outcome vector **y** (length *n* ) on the *CCR5*Δ*32* genotype dosages **g**, i.e.,

$$y=1\mu +g\beta_{\mathrm{obs}}+e$$

where μ is the phenotypic mean parameter for *CCR5* wildtype homozygotes, *β* *_obs_* is the allelic substitution effect of the *CCR5*Δ*32* deletion on the observed scale, and **e** is the residual vector. When conducting the GWAS, covariates including sex, age, age^2^, sex × age, and sex × age^2^ were fitted to reduce residual variance, and the first 20 principal components of the genomic kinship matrix were also fitted to remove the confounding effect due to population structure. The analysis was performed on 361,194 quality-controlled individuals, with restriction to samples of white British genetic ancestry. The detailed pipeline can be found at https://github.com/Nealelab/UK_Biobank_GWAS.

In order to assess the odds ratio estimates of the *CCR5*Δ*32* deletion, we transformed the estimated genetic effect from the observed scale ${\hat{\beta }}_{\mathrm{obs}}$ to its logistic scale $\hat{\beta }$. Typically, the phenotypic variance explained by the genetic variant is a very small fraction, and then ${\hat{\beta }}_{\mathrm{obs}}$, the disease prevalence, and the variant’s allele frequency together form a set of sufficient statistics for $\hat{\beta }$, making such transformation feasible (see Pirinen et al., 2013, formula 3.2, and an implementation in **Supplementary Table 1**). This provided the odds ratio of *CCR5*Δ*32* for each of the 131 disease phenotypes (**Supplementary Table 1**). Due to the lack of recorded HIV infection incidence in UK Biobank, we re-analyzed the contingency table in Samson et al. (1996), where the effect of natural *CCR5*Δ*32* deletion was first reported, to examine the odds ratio on HIV-1 infection in the Caucasian population. Estimated from a logistic regression, the odds ratio of a *CCR5*Δ*32* substitution is 0.56 (*p* = 1.03 × 10^−4^), though *CCR5*Δ*32* homozygotes appear to be completely immune to macrophage- and dual-tropic HIV-1 strains (Samson et al., 1996).

**Figure 1:**
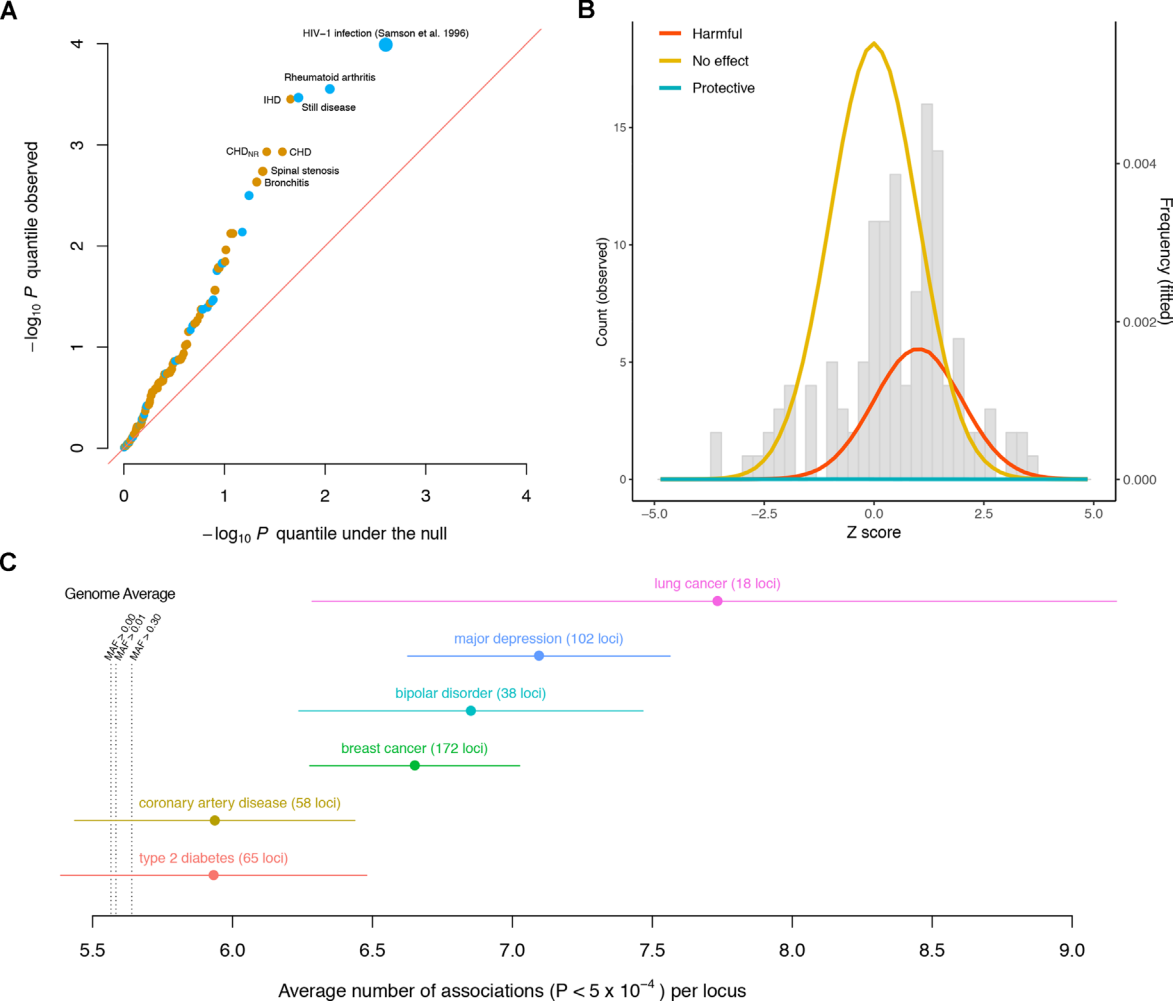
Pleiotropic nature of disease-associated variants. **(A)** Quantile–quantile plot of the −log_10_ *p* values of the association between the *CCR5*Δ*32* variant and different diseases, including 131 GWASed curated disease phenotypes in the UK Biobank and HIV-1 infection from Samson et al. (1996). Diseases with false discovery rate < 5% are labeled. Phenotypes for which the *CCR5*Δ*32* deletion elevates/reduces their risks are colored orange/blue, respectively. The sizes of the dots are proportional to the magnitudes of the deviations of their odds ratios from one. The red diagonal line represents equality between the *x* and *y* axes. **(B)** Distribution of *CCR5*Δ*32* effects on complex diseases. The genetic effects are shown as *Z* scores, i.e., standardized by their standard errors. The gray histogram shows the observed distribution of *Z* scores across 131 curated disease phenotypes in UK Biobank. The colored densities give the fitted mixture distribution, consisting of three Gaussian components, where the estimated proportion of harmful effects is about 23%. **(C)** Pleiotropy of the established loci for six diseases is evaluated by the number of associations per locus in Neale’s lab round 1 UK Biobank GWAS. Each dot represents the average number of associations per locus for the corresponding disease, and the whiskers represent standard errors. MAF: minor allele frequency.

The observed *p* value distribution across the diseases significantly deviates from what we expect under the null, indicating that the variant has effects on a significant subset of the diseases (**Figure 1A**). For instance, the *CCR5*Δ*32* variant has significant effects (false discovery rate < 5%) on rheumatoid arthritis (RA), Still disease (SD), ischemic heart disease (IHD), coronary heart disease (CHD), CHD with no revascularizations (CHD*_NR_*), spinal stenosis (SS), and bronchitis. Notably, among these seven diseases, the effects of the *CCR5*Δ*32* deletion on autoimmune (RA and SD) and other (IHD, CHD, CHD*_NR_*, SS, and bronchitis) diseases have opposite directions.

From the estimated odds ratios, regardless of statistical significance, the *CCR5*Δ*32* deletion appears to elevate the risk for 93 out of the 131 disease phenotypes in UK Biobank, versus the other 38 where the deletion appears to be protective against the diseases (**Supplementary Table 1**). This is notably enriched for harmful effects (*p* = 1.55 × 10^−6^, Wilcoxon signed rank test with continuity correction) if assuming the diseases are independent for simplicity. As most of these associations could be statistically zero according to the current data, in order to more stringently estimate the proportion of harmful effects across these diseases, we modeled the 131 GWAS *Z* scores as drawn from a mixture distribution of

$$\pi_{-}N(-\mu_{Z},1)+\pi_{0}N(0,1)+\pi +N(\mu_{Z},1).$$

Via a full likelihood estimation and bootstrapping (code available at https://github.com/xiashen/ccr5delta32), we estimated the proportion of harmful effects ${\hat{\pi }}_{+}=0.231\;(p=1.6\times 10^{-7})$, null effects ${\hat{\pi }}_{0}=0.769\;(p=2.1\times 10^{-11})$, protective effects ${\hat{\pi }}_{+}=0.000\;(p=0.9999)$, and the average harmful effect *Z* score ${\hat{\mu }}_{Z}=1.003\;(p=4.0\times 10^{-3})$. This is equivalent to about 30 out of the 131 diseases having elevated risk due to the Δ32 mutation, while comparatively, the mutation’s protective effect is nearly none (**Figure 1B**).

### Established Disease Susceptibility loci Tend to be Pleiotropic

It is arguable that the *CCR5*Δ*32* deletion might happen to be a special case, showing substantial pleiotropic effects on a wide range of phenotypes. How about potential gene editing for other diseases? Here, based on established disease-associated variants, we try to examine the likelihood that gene editing would result in side effects on other phenotypes.

In order to extend the consideration of pleiotropic effects to complex diseases in general, we investigated discovered susceptibility loci for six severe diseases in human population: breast cancer (Michailidou et al., 2017), lung cancer (McKay et al., 2017), coronary artery disease (CAD) (Nikpay et al., 2015), type 2 diabetes (T2D) (Morris et al., 2012), bipolar disorder (BIP) (Stahl et al., 2019), and major depressive disorder (MDD) (Howard et al., 2019). Again, in a different manner, we used the publicly available UK Biobank GWAS results by Neale’s lab (http://www.nealelab.is/uk-biobank). Each SNP was quantified for its number of associations across all the phenotypes (*p* < 5 × 10^−4^). The genome average of this quantity was 5.56 associations per SNP for all the variants (median = 5). Even for the variants with minor allele frequency larger than 0.3, the average number of associations was 5.64 per SNP (median = 5). For every disease among the six, the average number of associations of its reported susceptibility loci was larger than the genome average (**Figure 1C**, **Supplementary Table 2**). The results indicate that pleiotropic effects are ubiquitous and even enriched for many established loci associated with complex diseases.

## Discussion

Starting from the *CCR5*Δ*32* deletion, a site targeted by He Jiankui in his gene editing surgery, we investigated the pleiotropic nature of this deletion and some other disease-associated variants, using massive publicly available GWAS results from the UK Biobank. The results highlight that pleiotropy should always be carefully considered before gene editing treatment for han complex diseases.

Our results suggest that, in He Jiankui’s CRISPR experiment, even if the surgery does produce a deletion effect the same as *CCR5*Δ*32*, the treated girls would be prone to an elevated risk of cardiovascular and other potential diseases. It also seems true that the surgery would be more harmful than beneficial, considering the number of diseases that it might have effects on. Some of these diseases are not only common, but also essential contributors to the mortality rate of the current human population (Timmers et al., 2019). Although there is criticism about Wei and Nielsen (2019)’s pipeline, regardless of the level of statistical significance in their analysis, our additional results here do provide evidence that the Δ32 mutation’s potential effect on mortality may be related to its side effects on other more common diseases.

Besides the issue with pleiotropy, gene editing in humans may lead to other unwanted consequences. Although the CRISPR-Cas9 technology has been shown to be a reliable method to introduce mutations to the target site, it appears that He Jiankui has also ignored the possibility of any off-target effects that might be induced in the process (Zhang et al., 2015). Furthermore, from an evolutionary perspective, we should be careful before introducing any artificial mutation to the human gene pool, even if the introduced mutation might have negligible side effects for the population. For instance, as it is likely that the introduced mutation is in linkage disequilibrium with another functional variant under positive selection, due to genetic hitchhiking (Barton, 2000), the introduced mutation can gain allele frequency so that its effects on the population are revealed. However, we do not suggest a complete ban of gene editing treatments. Similar to the development of any treatment, what is essential is the trade-off between positive and negative effects. One can imagine that a gene editing surgery removing a severely impactful monogenic mutation could be valuable to certain individuals, given that the side effects are known to be none or so small that they do not matter compared to the monogenic disease itself. Unfortunately, for most complex diseases, the situation does not appear to be as straightforward at all.

Pleiotropy, i.e., a gene or genetic variant having complex effects on various phenotypes, is a very common phenomenon. It is encouraging to foresee the potential of gene editing in humans as treatments for diseases. However, practitioners such as He Jiankui had uninformed opinions towards *CCR5*Δ*32*’s effect against HIV and showed disrespect to the complexity of genome biology resulting from billions of years of evolution. The data presented here were all publicly available, sufficient to prevent anyone from even considering the experiment on living human embryos. Unfortunately, all these established resources were overlooked. We provided additional evidence to evaluate He Jiankui’s actions and to guide considerations in future gene editing research, as it undeniably is a field with great potential.

## Data Availability

The datasets analyzed in this study can be found in the Supplementary Tables and references.

## Author Contributions

XS initiated and coordinated the study. TL and XS performed data analysis. Both authors contributed to writing the paper.

## Funding

XS was in receipt of funding from the Recruitment Program of Global Experts in China and a Swedish Research Council grant (No. 2017-02543).

## Conflict of Interest Statement

The authors declare that the research was conducted in the absence of any commercial or financial relationships that could be construed as a potential conflict of interest.
